# Dietary practices and associated factors among adult diabetic patients at academic tertiary-level hospitals in central Ethiopia: convergent mixed study

**DOI:** 10.1186/s40842-024-00192-7

**Published:** 2024-10-30

**Authors:** Sentayehu Admasu Saliya, Taye Mezgebu Ashine, Asnakech Zekiwos Heliso, Getachew Ossabo Babore, Bethelhem Birhanu, Awoke Girma Hailu, Sisay Foga Sebro, Elias Ezo, Selamawit Wondale Begizew

**Affiliations:** 1 Department of comprehensive Nursing, College of Medicine and Health Science, Wachemo University, Hosanna, Ethiopia; 2 Emergency Medicine and Critical Care Nursing, College of Medicine and Health Science, Wachemo University, Hosanna, Ethiopia; 3 Department of Pediatric and Child Health Nursing, College of Medicine and Health Science, Wachemo University, Hosanna, Ethiopia

**Keywords:** Diabetes mellitus, Dietary practice, Central Ethiopia

## Abstract

**Background:**

Diabetes mellitus (DM) is one of the most prevalent chronic diseases worldwide. Despite the existence of national strategies to prevent potential complications, DM-related morbidities and mortality continue to rise in Ethiopia. Although studies have been conducted regarding dietary practices among DM patients in the country, there is a lack of in-depth understanding of the situation.

**Objective:**

To assess dietary practices and associated factors among adult DM patients at academic tertiary-level hospitals in central Ethiopia in 2024.

**Methods:**

A concurrent mixed-methods study was conducted from January 01 to 30, 2024, involving 420 adult DM patients. Participants were selected using a simple random sampling technique for the quantitative study. A purposive sampling technique was used to select sixteen participants for qualitative analysis. For the quantitative study, a self-administered structured questionnaire was used to collect data, and in-depth interviews were conducted for the qualitative part. Multivariate binary logistic regressions were used to assess the explanatory variables associated with dietary practice. A thematic analysis was performed for qualitative data.

**Result:**

Of 420 eligible participants, 406 (96.7%) participated in the study. The overall proportion of good dietary practices among participants was 172 (42.4%). Being female, residing in urban areas, having a family history of DM, and having good dietary knowledge were significantly associated with better dietary practices. From the qualitative analysis, two themes emerged "Living as before and not adhering to diabetic dietary recommendations" and "Barriers to adherence to effective eating practices."

**Conclusions and recommendations:**

Less than half of adult DM patients had good dietary practices. Sex, geographic location, family history of DM, and level of knowledge of diabetic diet were associated with dietary practice. Intervention programs on awareness creation and training to improve the dietary practice by stakeholders were recommended.

## Introduction

Diabetes mellitus (DM), a set of metabolic disorders, is characterized by persistent high blood glucose levels because of errors in insulin secretion, its action, or both [1]. The dietary practices of patients refer to their food choices, guided by diabetes nutrition education that emphasizes consuming foods low in fat, high in fiber, and low in sodium [2]. Good dietary practices are considered fundamental in diabetes treatment [3].

The World Health Organization (WHO) recommends that patients maintain a healthy body weight, engage in regular physical exercise for at least 30 min of moderate-intensity activity per day, follow a nutritious diet, avoid tobacco use, and achieve and maintain a healthy body weight [4]. The American Diabetes Association (ADA) suggests consuming fruits, vegetables, whole grains, legumes, and r-containing foods, and minimizing high-sucrose foods for the secondary prevention of Type 2 Diabetes [5].

Diabetes is currently one of the most prevalent non-communicable diseases globally, with significant associated complications. Reported diabetic complications include retinopathy (2.7–25%), neuropathy (4.8–35%), kidney disease (18.2–23.2%), hypertension (23–54.8%), and depression (13–61%)(13–61% [6].

In Africa, the prevalence of diabetes and its related mortality and disability are rising. Sedentary behavior, rapid urbanization, and dietary changes are expected to quadruple the prevalence of diabetes mellitus in the next 25 years [7, 8].

According to estimates from the International Diabetes Federation (IDF), Ethiopia had 2.57(5.2%) adult people aged 20–79 years with diabetes, making it the largest diabetes population in sub-Saharan Africa [9].

A systematic review in Ethiopia revealed a diabetes mellitus prevalence ranging from 2.0% to 6.5% [10]. Previous studies in Ethiopia reported varying rates of poor dietary practices: 40.6% at Arba Minch General Hospital, Gamo Zone [11], 53.8% in Dire Dawa [12], and 64% in Bahir Dar City [13].

Various studies have shown that dietary practices in diabetic patients are strongly associated with factors such as nutrition education [14], education level, family support, gender, age, presence of comorbidity, income, employment status, disease knowledge, and dietary advice quality [15, 16].

While previous studies have focused on quantitative findings in specific areas, this study aims to provide a deeper understanding of dietary practices and associated factors among adult patients with diabetes mellitus in central Ethiopian tertiary hospitals in 2024 by combining quantitative and qualitative approaches.

## Methods and materials

### Study approach

The study was conducted using a convergent mixed method approach from January 01–30 / 2024.

### Study area

The study was conducted in three tertiary hospitals in central Ethiopia, namely Wolikite University Hospital, Wachemo University Hospital, and Worabe Hospital.

Wolikite University Hospital, situated 172 km south of Addis Ababa in Wolikite town, was established in 2018 as a teaching hospital for health science students. The hospital provides preventive, curative, and rehabilitative clinical services across outpatient, inpatient, emergency, and maternal and child health departments. Specifically, the hospital's outpatient department includes two Diabetes Mellitus follow-up OPD rooms for regular patient monitoring, currently managing 1150 diabetic patients, with 690 diagnosed with Type II Diabetes Mellitus [17].

Worabe Hospital, located in Worabe town, 172 km south of Addis Ababa, offers services in outpatient, obstetrics and gynecology, internal medicine, and surgery. The hospital's outpatient department houses a diabetic follow-up clinic, overseeing around 900 DM patients, with 610 diagnosed with Type II DM.

Wachemo University Hospital referral, positioned in Hosanna town, 232 km south of Addis Ababa, supports 18 outpatient departments and serves as a teaching hospital. The hospital provides preventive, curative, and rehabilitative clinical services across various departments, including outpatient, inpatient, emergency, and maternal and child health. The Diabetes Mellitus follow-up clinic in the outpatient department manages 1300 diabetic patients, with 890 diagnosed with Type II Diabetes Mellitus.

#### Populations

The source population for this study includes all adults diagnosed with diabetes mellitus undergoing regular follow-up at the diabetes clinic. The study population comprised randomly selected adult patients with diabetes mellitus who met the inclusion criteria and had regular follow-ups, forming the basis for both the quantitative and qualitative studies. Inclusion criteria for the quantitative study involved diabetes patients over 18 years who had at least one prior follow-up before the data collection period, while for the qualitative study, patients diagnosed with diabetes mellitus under 18 years, with an illness duration of less than 6 months, willing to participate, and providing informed voluntary consent were included. Exclusion criteria encompassed mentally incompetent individuals and those with communication challenges, while the qualitative study excluded pregnant women, patients with known mental issues, communication difficulties, and severe illnesses.

### Sample size determination and sampling techniques

To determine the sample size for the quantitative study, we considered the outcome variables and used the prevalence of poor dietary practices (*p* = 53.7%) reported by [18] to estimate the minimum sample size needed to achieve the study's objective. We then calculated the sample size using a single population proportion formula, considering the following assumptions: a 95% confidence level with a corresponding value of 1.96 for normal distribution and an absolute precision of 5%. The following statistical formula was used:

$$n=\frac{{\text{Z }(\upalpha/2)}^{2}\text{P}(1-\text{P})}{{\text{d}}^{2}}$$

*n* = the minimum sample size needed,

*p* = % 53.7% prevalence of poor dietary practice in a similar study.

z = the standard value for normal distribution with a confidence level of α = 95%

d = the error margin between the sample and the population (0.05).

$$\text{Z}=1.96,\text{ P }=53.7\text{\%},\text{ d }= 5\text{\% }=0.05,\text{ n }=\frac{{(1.96)}^{2}\times 0.537 (1-0.537)}{{(0.05)}^{2}}=382$$

Add 10% by considering the non-response rate, which is equal to 420.

The sample size of the specific objective was determined considering significantly associated factors with the two-sided confidence level of the outcome variable of 95%, power 80%, and the ratio of exposed to unexposed 1:1 using Epi Info Version 7.2.2.6 and the calculated sample size for the selected factors are shown in the table below (Table 1).

**Table 1:** Sample size determination to conduct a study on dietary Practices and associated factors

Significantly associated factors of dietary Practices of DM patient	Reference	% of non-exposed	%of exposed	% CI	Power	Allocation	Total sample size
Food security status AOR( 5.3 (2.8–9.9))	[11]	28.9	57.6	95	80	1:1	106
Nutrition education AOR( 2.2 (1.1–4.6))	[11]	38.7	55.0	95	80	1:1	316
Age category(18–32) AOR 6.05(2.44–15.03)	[19]	16.9	61.9	95	80	1:1	44
Monthly income AOR(0.15(0.09–0.27))	[19]	18.0	55.6	95	80	1:1	60
Duration of DM AOR (4.16(1.85–9.37))	[19]	24.4	40.2	95	80	1:1	298

Finally, the required sample size for this particular study was decided by taking the maximum sample size (i.e. first specific objective = 382) from the calculated sample size and then adding 10% considering the non-response rate, therefore, 420 samples of study participants required to carry out this particular study.

For the qualitative study, the sample size was determined based on data saturation A total of 16 participants were included in the qualitative study.

Sampling for the quantitative study was carried out after obtaining the average three-month number of patients with DM who have follow-ups in tertiary hospitals. The total sample size was distributed proportionally according to the number of diabetic patients at follow-up in each academic tertiary hospital.

A systematic random sampling technique was used to select study subjects from tertiary hospitals based on patient flow during the study period. The sampling interval was obtained by dividing the sampling proportion in three three-month average numbers (N) by the number of samples (n). The K-value, representing the interval for patient selection, was determined by dividing the total number of type-2 diabetic patients in Wachemo University Referral Hospital (890 patients), Wolikite University Referral Hospital (690 patients), and Worabe Comprehensive Hospital (610 patients) by the respective allocated proportions for each hospital (171, 132, and 117). This method ensured a systematic approach to patient selection, with every 5th patient being chosen for inclusion in the study. The initial sample for each hospital was selected randomly from the range of 1 to K, ensuring a fair and unbiased representation of patients across the different healthcare facilities.

The first participant (starting individual) was selected using the simple random sampling technique, and then other study participants were selected through every K interval using the systematic sampling technique until the desired sample size.

A purposive sampling strategy was used to select study participants for qualitative study. Qualitative data were obtained from the same existing patients from the quantitative sample. Sampling and data collection were continued until data saturation was reached and no new data was obtained from the interviews.

#### Study variables

Dependent variable

- ❖ Dietary Practice of the patient with DM

Independent variable

- ❖ Socio-demographic: Age, sex marital status, educational status, residence, occupational status, and income
- ❖ Health and information related:—Duration of DM, Presence of comorbidity, nutritional education, physical exercise, regular follow-up, substances, and food choices.

#### Data collection procedure

Data for the quantitative study were collected by three nurse professionals under the supervision of the principal investigator. The data collectors received a one-day training from the principal investigator on the study's purpose, tools, and sampling methods. After obtaining written informed consent from study participants, data were collected by the data collectors through structured questionnaires administered via interviews.

For the qualitative study, one-on-one interviews were conducted in Amharic in a quiet, private, and distraction-free environment where study participants felt comfortable. The interview sessions lasted between 25–30 min. With the participants' written consent, the primary investigator recorded the interviews and took notes to capture the original accounts of the participants' responses. On the same day, each interview was transcribed and translated into English by crosschecking both the audio record and the notes. The transcripts of each interview were read and reread to gain an understanding of the whole situation and then reread slowly to determine its significant features.

### Operational definition

*Regular exercise*: 20 min—30 min of aerobic exercise such as walking or swimming 3–4 days per week [19].

*Good knowledge*: when patients respond to the mean or above the mean score on knowledge questions [19].

*Poor knowledge*: when patients respond below the mean score on knowledge questions [19].

*Good dietary practice*: when patients respond to the mean or above the mean score on practice question [20].

*Poor dietary practice*: when patients respond the below the mean score on practice question [20].

*Comorbidity*: having one or more chronic diseases in patients living with DM [21].

#### Instrument

The questionnaire administered by the interviewer was adapted from different literature on the dietary Practices of DM patients [11, 12]. The tools contain 42 questions, which were designed to cover the following sections; participant’s socio-demographic characteristics, Health profile-related questions, knowledge measurement questions, and dietary Practices measurement questions of patients with DM.

To assess Health profile-related questions, 10 items were used. the tool's reliability and in the current study was tested using the data from the study data, and Cronbach's alpha value was 0.961.

To asses’ knowledge of participants’ diabetic diet were described in seven on knowledge question were used. In a previous study, Cronbach's alpha was recorded as 0.65 [22], the tool's reliability in the current study was tested using the data from the study data, and Cronbach's alpha value was 00.83. A visual examination of the histogram revealed that the knowledge scores was approximately normally distributed and had a skewness of -0.372 (SE = 0.121) and a kurtosis of -0.529 (SE = 0.241), indicating it is normally distributed. After reverse scoring for one negative items (item 7), the overall mean was calculated. Respondents who scored more than the mean on the total of all knowledge related questions were categorized as having a “Good knowledge” otherwise “poor knowledge”.

To assess dietary Practices measurement questions of patients with DM was used. The general dietary guidelines of the South African Diabetes Association and Amalmal Worku's modified version of the fifteen Morisky medication adherence scale (MMAS-8) were used to produce the questionnaires used to evaluate the dietary practices of Dm patients [23]. It comprised fifteen components, and the mean value was used to categorize the respondents' dietary practices into good and poor categories. Each of the items contains two response options (Yes = 1and No = 0, here yes was used for those responses which were negatively answered or far true answer from what science is talking about). As a result, by selecting Yes or No for each statement, respondents were able to select the correct response. Ultimately, the variables related to dietary practice were calculated and scores were assigned to the respondents' dietary practices. In a previous study, Cronbach's alpha was recorded as 0.65 [22]. After reverse scoring for one negative items (item 5, 6, and 11), the overall mean was calculated. The mean score was calculated after checking the normality of the distribution. A visual examination of the histogram revealed that the dietary Practices measurement questions of patients with DM. scores was approximately normally distributed and had a skewness of 0.034 (SE = 0.121) and a kurtosis of 0.175 (SE = 0.241), indicating it is normally distributed. Respondents who scored more than the mean were considered “good dietary Practices” otherwise “poor dietary Practices”.

In-depth interviews were conducted to collect qualitative data. The interview was guided by an in-depth interview guide developed from the existing academic literature and a discussion among the principal investigator and immediate supervisors. Purposive sampling was used to choose participants for in-depth interviews and conducted face-to-face interviews with participants. An interview guide was utilized to outline open topics. The interview contained open-ended questions that covered socio-demographic characteristics. What do you think about the role of a diabetic diet in controlling your blood sugar level? Have you received information from your healthcare provider on the importance of adhering to dietary modifications? Are you able to adhere to dietary modifications?

#### Data quality control

To achieve the quantitative data quality control issue of quantitative data; training was given to data collectors and supervisors using the local language (Amharic). The questionnaire was translated into the local language for data collection and then retranslated back into the English version to check for consistency. A pre-test was conducted at Sodo Comprehensive Specialized Hospital to ensure its consistency and errors were corrected. Short-term discussions were held after each data collection day with all data collectors and supervisors to address challenges and to make them clear on how to solve the challenges they faced. The completeness of each questionnaire was checked by the principal investigator and supervisors daily.

After being recorded in Amharic, the qualitative data were transcribed and analyzed in English. The QDA (qualitative data analysis) miner software was used to enter and analyze the qualitative data that had been transcribed. Thematic analysis was performed using an inductive approach.

To ensure the credibility of the study; sustained interaction with participants and to preserve data source triangulation, in-depth research was conducted in a confidential and comfortable setting among those participants. To ensure the dependability of the study; the audio tape was transcribed in Amharic word by word before being translated into English by cross-checking both the audio record and the notes. Transcripts of each interview were read and reread to gain an understanding of the whole situation and then reread slowly to determine its significant features. To ensure the conformability of the study; an interview guide was used and the semi-structured interview guide allowed focus and flexibility during the interview by coding the participants' own words instead of the researchers' opinions. To ensure the transferability of the study; purposive quota criteria and data saturation during the interview were used.

### Data processing and analysis

The data was initially cleaned and coded before being entered into Epi-data version 4.6 software and subsequently exported to SPSS version 26 for analysis. Various dummy tables, graphs, and descriptive summaries were employed to present the study variables. The Hosmer and Lemeshow goodness-of-fit test was utilized to evaluate the model's fitness. Following this, logistic regressions was conducted to assess the strength of the association between each independent variable and the outcome variables. All variables with *p*-values less than 0.25 in bivariate logistic regressions were fitted into the backward stepwise multivariable logistic regression model. Finally, only those independent variables that maintain their association with outcome variables in multivariate regressions (*p*-value < 0.05) were declared statistically significant. The odds ratio with its *p*-value and confidence interval was used or reported in each logistic regression analysis. To measure the strength of the association between the outcome and the independent variables, the crude odds ratio (COR) and the adjusted odds ratio (AOR) along with a 95% confidence interval (CI) were calculated.

## Result

### Quantitative result

#### Socio-demographic characteristics

Of 420 eligible DM patients, 406(96.7%) participated in the study. More than half 225(55.4%) of the participants were male. The mean age (± SD) of the participants was 42.07 ± (11.525) years with an age range from 23 to 78 years. The majority of the study participants (55.7%) were less than the age group of 40 years. Of the participants, two-thirds 265 (65.3%) were Hadiya in their ethnicity, and more than two-thirds 283(69.7%) of the participants were protestant religious followers (Table 2).

**Table 2:** Socio-demographic characteristics of adult patients with diabetic mellitus

Variables	Category	Frequency	Percentage
Age	< 40	226	55.7%
	40–60	150	36.9%
	> 60	30	7.4%
Sex	Male	225	55.4%
	Female	181	44.6%
level of education	unable to read and write	72	17.7%
	able to read and write	151	37.2%
	Primary school	40	9.9%
	Secondary school	45	11.1%
	College graduate or above	98	24.1%
marital status	Single	99	24.4%
	Married	277	68.2%
	Divorced	8	2.0%
	Widowed	22	5.4%
Occupation	Farmer	70	17.2%
	Government employee	132	32.5%
	Merchant	22	5.4%
	private employ	43	10.6%
	daily laborer	12	3.0%
	housewife	127	31.3%
Ethnicity	Hadiya	265	65.3%
	Kambata	105	25.9%
	Silte	20	4.9%
	Gurage	16	3.9%
Religion	Protestant	283	69.7%
	Orthodox	82	20.2%
	Muslim	37	9.1%
	Catholic	4	1.0%
Residence	Urban	295	72.7%
	Rural	111	27.3%
Income	< 5000	138	33.9.0%
	5001–7500	129	31.77%
	7501–10000	99	24.4%
	> 10,000	40	9.93%
Family size	< 3	79	19.5%
	3–6	217	53.4%
	> 6	110	27.1%

#### Health and information-related profile

More than half 245(60.3%) of the participants had diabetic duration > 36 months and one-third 135(33.3%) of them were on follow-up for between 12–36 months. One hundred twenty-six (31%) and 170(41.9%) participants had comorbidity and family history of diabetes mellitus, respectively (Table 3).

**Table 3:** Health-related profile of adult patients with diabetic mellitus on follow-up

Variables	Category	Frequency	Percentage
Duration of diseases in month	< 12	22	5.4%
	12–36	139	34.2%
	> 36	245	60.4%
Follow-up duration in month	12	35	8.6%
	12–36	135	33.3%
	36–60	121	29.8%
	> 60	115	28.3%
Comorbidity	Yes	126	31.0%
	No	280	69.0%
Types of comorbidity	Hypertension	86	21.2%
	Hyperlipidemia	12	3%
	heart failure	28	6.9%
Family history of DM	Yes	170	41.9%
	No	236	58.1%
Way of treatment modalities	Insulin	302	74.4%
	Tablet	96	23.6%
	controlling diet	4	1.0%
	insulin, tablet, and diet	4	1.0%
Have made a change of dietary habit after diabetics	Yes	345	85.0%
	No	61	15.0%
If yes for dietary habit change, what is source of information?	Media	27	6.7%
	Health professionals	235	57.9%
	health professionals and media	62	15.3%
	diabetic patients and health professionals	26	6.4%
participated in a regular exercise	3 times a week for 30 min)	44	10.8%
	less than 3 times a week)	97	23.9%
	Never	265	65.3%

#### Barriers to following a dietary plan

**Fig. 1:**
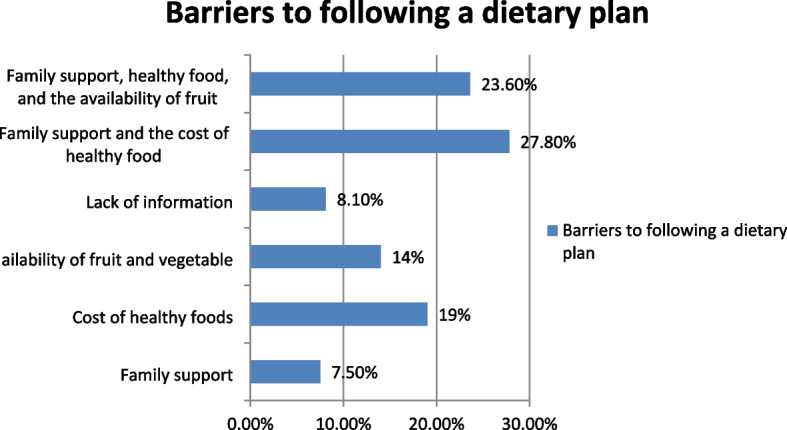
Barriers to follow a dietary plan

Regarding barriers to following the dietary plan, family support (7.4%), cost of healthy food 113 (27.8%), and availability of fruit 57 (14%) were the major ones (Fig. 1).

#### Proportion of diet knowledge of diabetic patients

**Fig. 2:**
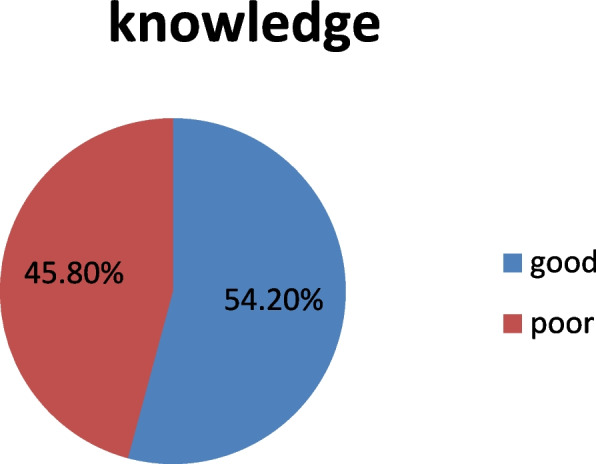
Proportion of dietary knowledge of adult patients with diabetic mellitus

The overall proportion of good diet knowledge among diabetic patients was 45.8%( Fig. 2).

#### Proportion of dietary practice of diabetic patients

Among our study participants, 38.9% reported having a planned meal ahead, 43.6% followed a flexible meal plan, and only 41.6% consumed vegetables more than three times a day. On the contrary, 24.1% of the participants adhered to a specific diet plan and 4.2% consumed fruit more than three times a day (Table 4).

**Table 4:** The dietary practice of adult patients with diabetic mellitus

Variables	Response	Frequency	Percentage
Do you plan meals and eat ahead	Yes	158	38.9%
	No	248	61.1%
Took a meal based on the dietary plan yesterday	Yes	126	31%
	No	280	69%
Took meals based on a dietary plan over the past 2 weeks	Yes	122	30%
	No	284	70%
Always eat based on your dietary plan	Yes	98	24.1%
	No	308	75.9%
Never feel stressed to stick on dietary plan	Yes	165	40.6%
	No	241	59.4%
Have no feelings of dietary deprivation	Yes	212	52.2%
	No	194	47.8%
Did you follow the flexible eating plan	Yes	176	43.3%
	No	230	56.7%
How many times in a day do you eat?	one time	16	3.9%
	two times	33	8.1%
	three times	329	81%
	more than three times	28	6.9%
Do you take your meal at the appropriate time?	Yes	358	88.2%
	No	48	11.8%
Which one do you use for cooking	Saturated fatty acid	296	72.9%
	Unsaturated fatty acid	110	27.1%
Do you drink sweets and beverages regularly	Yes	39	9.6%
	No	367	90.4%
Do you take the fat of meat regularly	Yes	27	6.7%
	No	379	93.3%
Do you consume fruits ≥ 3/a day	Yes	17	4.2%
	No	389	95.8%
Do you eat vegetables ≥ 3/a day	Yes	170	41.9%
	No	236	58.1%
How do you usually take fruits	Whole fruit	333	82%
	Juiced	73	18%

**Fig. 3:**
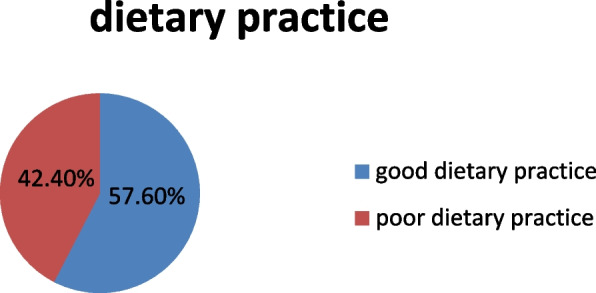
Proportion of dietary Practice of adult patients with diabetic mellitus on follow-up

The overall proportion of good dietary Practice among diabetic patients was 172 (42.4% %) [95% CL (37.7–47.5)], see (Fig. 3).

#### Factors associated with the dietary practice of diabetic patients

Bivariate logistic regression analysis was used to evaluate various independent variables with outcome variables. Thus, marital status, sex, residence, family size Family history of DM, comorbid diseases, and knowledge were significantly associated with the diet Practice of adult diabetic patients at follow-up. Significantly associated with the diet Practice of adult diabetic patients at follow-up were all variables with *P*-values less than 0.25 that were fitted into the backward likelihood multivariate logistic regression model.

However, on multivariable analysis; sex, residence, family history of DM, and knowledge were significantly associated with the diet Practice of adult diabetic patients at follow-up. The probability of female patients having good diet practice was 2.22 times higher than that of male patients (AOR: 2.22, 95% CI 1.283–3.841). Furthermore, the odds of good dietary Practice among urban diabetic patients were 3.136 times higher than those of rural areas (AOR: 3.136, 95% CI 1.666–5.904). Additionally, individuals with a family history of diabetes had 3.014 times higher odds of having good dietary Practices compared to those without a family history of diabetes (AOR: 3.014, 95% CI 1.654–5.492). Furthermore, diabetic patients with good knowledge had 2.876 times higher odds of having a good diet compared to those with poor knowledge (AOR: 2.876, 95% CI 1.524–5.428) (Table 5).

**Table 5:** Bivariate and multivariate analysis of the dietary practice of adult patients with diabetic mellitus on follow-up

Variables		Dietary Practice		COR(95%CL)	AOR (95%CI)
		Good	Poor		
Sex	Female	79	132	1.099(0.740–1.633)	2.220 (1.283–3.841)**
	Male	93	102	1	1
marital status	Single	50	49	4.592 (1.450–14.543)	1.884(1.034–3.432)
	married	114	163	3.147(1.038–9.545)	1.741(0.909–2.381)
	Divorced	4	4	4.500(0.775–26.133)	1.271(0.978–1.651)
	Window	4	18	1	1
Follow-up duration in month	12	26	9	6.090 (2.595–14.292)	6.241(2.45–15.895)
	12–36	60	75	1.686 (1.005–2.831)	1.841(1.814–3.185)
	36–60	49	72	1.435(0.841–2.446)	1.313(0.739–2.333)
	> 60	37	78	1	1
Residence	Urban	144	151	2.827(1.740–4.593)	3.136 (1.666–5.904)*
	Rural	28	83	1	1
Family history of diabetes	Yes	68	102	0.846(0.567–1.263)	3.014 (1.654–5.492)***
	No	104	132	1	1
Comorbid diseases	Yes	37	89	0.447(0.285–0.70)	(0.534–1.994)
	No	135	145	1	1
Knowledge	Good	102	84	2.602(1.736–3.901)	2.876 (1.524–5.428)***
	Poor	70	150	1	1

*AOR* Adjusted odd ratio, *COR* Crude odd ratio

*; *p* value < 0.05, **; *p* value < 0.025, ***;*p* value < 0.0001

### Qualitative results

#### Participants’ characteristics

Sixteen patients were recruited for the qualitative study. The mean (± SD) age of the participants was 29.185 (± 3.67). The participants’ ages ranged from 25 to 67 years. Regarding their working positions, four were government employees, three were merchants, two were housewives and seven were farmers.

#### Living as before and not adhering to diabetic dietary recommendations

The majority of participants acknowledged the significance of diet in controlling blood glucose levels. Nearly all participants described their typical meal as consisting of injera. However, most participants did not recognize diabetic-friendly meal plans. This is evident from the following quotes:

“I do not want to say on every occasion, 'I am diabetic, I don't eat this.' That is too boring from the day of diagnosis, I have not changed my meals. I eat like everybody else. I don't want to be too picky about my meals." (37 years old male Participant 9).

“What does it matter if I avoid a meal that has sugar? I don't like to stress myself too much about what I eat, drink, and the like. When I am hungry, I eat whatever is in front of me. " (42 years old male Participant 1).

"Sugar for diabetic patients? No, we don't do that… Even I forget, my wife reminds me not to touch anything that has sugar. My problem is I don't plan my meals… this time this food and the like." (54 years old male Participant 14).

#### Barriers to adherence to effective diabetic dietary practice

A diverse array of responses emerged concerning obstacles to effective dietary practice adherence.

#### Societal pressure

Almost all respondents mentioned that societal pressure during gatherings sometimes affects their dietary practices. In Ethiopia, sharing food during gatherings is considered a way of expressing love.

As one participant expressed, "When I visit family or friends, people share food on a plate and insist that I join and have the food… they will say, 'Come, have a bite, we will pray. One bite can't kill you.'"(50 years old female Participant 16).

#### Accessibility

Many participants reported living in rural areas, resulting in reduced access to food variety, as illustrated by these quotations:

"Are you kidding? I am from a rural area. We don't have many options for fruits and vegetables. Even if we have the means and desire to purchase them, we must travel to the market, which occurs just once per week." (67 years old female Participant 6).

#### Lack of support

Participants highlighted the role of insufficient social support in hindering effective dietary practice adherence, as evidenced by the following statements:

"You know we are humans, and we require support from our families and communities as well. Sometimes I feel exhausted from my job (which requires a great deal of energy because I am a farmer). When I am hungry, I forget that I am diabetic and consume foods in front of me. We often need support and someone to remind us." (43 years old male Participant 15).

#### Knowledge

Lack of knowledge on dietary self-care practices such as exercise, proper nutrition, and blood glucose monitoring was cited as a barrier to implementing effective dietary practice adherence, as exemplified by these comments:

"No one has informed me about which foods to eat……This is my fourth year since my diagnosis of diabetes. I didn't realize that fruits and vegetables are important. I thought if I ate fruit, my blood glucose would rise." (50 years old female Participant 14).

Moreover, some participants indicated that strict adherence to effective dietary practices might be perceived as monotonous:

"As you can see, I am young, and I cannot stop everything at this age, especially food and drinks. How can I live without even sipping beer?….. Because I enjoy beer very much. I have continued consuming beer despite my diagnosis, although not excessively.” (60 years old male Participant 3).

## Discussion

This study aimed to investigate the dietary practices and associated factors among adult diabetes mellitus (DM) patients at academic tertiary-level hospitals in central Ethiopia. Through this institution-based cross-sectional study, the research delved into assessing and exploring the dietary practices of diabetic patients and the barriers that hinder their adherence to recommended dietary guidelines.

The study revealed that the overall proportion of good dietary practice among diabetic patients was 42.4% [95% CL (37.7, 47.5)]. As the finding indicates good dietary practice was low, this finding was reinforced by an in-depth interview where a participant expressed a tendency to continue living as before without adhering to diabetic dietary recommendations. While the majority of participants recognized the importance of diet in managing blood glucose levels, many described their typical meals as centered on Injera. Surprisingly, most participants did not identify diabetic-friendly meal plans as part of their dietary choices. The study revealed that the overall proportion of good dietary practice among diabetic patients was 42.4% [95% CL (37.7, 47.5)]. This finding is consistent with the findings from Gonder (46.7%) [24]. However, this finding is lower than studies conducted in other parts of Ethiopia: Addis Ababa (48.6%) [25], Hawassa (55.8%) [26], and Nigeria (76%) [27]. Differences in findings could be due to differences in the study setting, sample size, and socioeconomic status of participants. The other possible explanation could be the tool used to asses (measure outcome).

The proportion of good dietary practice among diabetic patients in this study is higher than the studies from; central Ethiopia 35.6% [21], Bahir Dar (35.9%) [13], and Dire Dawa, Ethiopia found a 53.8% prevalence of poor dietary practice [12]. The possible explanation for the variation could be due to differences in socioeconomic status of participants, study setting, and level of awareness.

The likelihood of female patients having a good dietary practice was 2.22 times higher than that of male patients. The finding is consistent with studies from; central Ethiopia [21], south-west Ethiopia [28], India [29] and Yemen [30]. One possible explanation for this finding is that women may be more receptive to changing their dietary habits than men. Additionally, as cooking is typically a task performed by women in Ethiopia, they may have more opportunities to prepare food per dietary recommendations for their health condition. This may contribute to the higher likelihood of good dietary practices among female patients compared to male patients. Contrasting these results, previous research conducted in Nepal [31] lower likelihood of good dietary practice among males than females.

The odds of good dietary practice among urban diabetic patients were 3.136 times higher than those in rural areas. This finding is consistent with studies from; Bahir Dar [24], Tigray Ethiopia [32], Yemen [30], and Gambella [33]. One possible explanation for this could be that diabetic patients living in urban areas have easier access to a variety of food and information, which may enhance their ability to practice dietary self-care management for diabetes [34]. This advantage in access to food and information is supported by in-depth interviews, as most participants explained that they reside in rural areas, where they have limited access to a variety of food. This disparity in access to food resources between urban and rural areas may significantly affect the dietary choices and self-care practices of individuals living with diabetes.

Individuals with a family history of diabetes had 3.014 times higher odds of having good dietary practices compared to those without a family history of diabetes. This finding is consistent with previous studies conducted in Dire Dawa [35] and western India [36]. One possible explanation for the association between family history and good dietary practices among diabetic patients is that individuals with a family history of diabetes may have a better understanding of dietary recommendations due to the availability of information from their family members who have experienced the condition. This may contribute to their increased likelihood of adhering to beneficial dietary practices. The finding was further reinforced by in-depth interviews, revealing that social support and societal pressure posed barriers to adhering to an effective diabetic diet. Nearly all respondents noted that societal pressure during gatherings could impact their dietary choices. In Ethiopia, the act of sharing food during social gatherings is a cultural expression of love. Participants emphasized the impact of inadequate social support on impeding adherence to effective dietary practices, as illustrated by the following statements.Diabetic patients with good knowledge had 2.876 times higher odds of having good dietary practice compared to those with poor knowledge. This finding is consistent with studies from central Ethiopia [21], Addis Ababa [37], Bahir Dar [38] and Arba Minch [11], Dire Dawa [35], Kenya [39]. A possible explanation for this could be knowledge of diabetic patients about diabetes self-care practices increase dietary practice, which is one of the components of diabetic self-care practice. Good dietary practice is the pillar for diabetes self-management, and poor dietary practices are associated with poor knowledge about diabetes and vice versa [31]. This was further corroborated by in-depth interviews, as some participants expressed their challenges in adhering to effective dietary practices due to a lack of awareness about dietary self-care, including exercise, diet, foot care, and blood glucose monitoring. Additionally, some participants described their difficulty in adhering to effective dietary practices due to the perceived monotony of strict adherence. These insights highlight the need for tailored education and support to address knowledge gaps and perceptions that hinder adherence to beneficial dietary practices among individuals with diabetes.

## Conclusion and recommendation

This study unveiled that less than half of diabetes mellitus (DM) patients exhibited good eating practices. Factors such as sex, place of residence, family history of diabetes, and level of dietary knowledge showed significant associations with dietary practices. Through qualitative analysis, notable barriers hindering compliance with beneficial dietary regimens were identified, including societal pressure, accessibility to foods, lack of support, and lack of knowledge. Our recommendations include advising patients to adhere to dietary recommendations, with a specific emphasis on male patients paying close attention to diabetic diets. Health professionals and healthcare facilities are encouraged to provide tailored health education based on geographic locations and involve the families of diabetic patients in these educational efforts. Lastly, families and communities should offer support to diabetic patients in managing their diets effectively.

## Strengths and limitations

### Strength

- ❖ The study used a mixed convergence method in which quantitative and qualitative data were collected and analyzed, and then the analyzed data were compared to see if the data confirmed or disproved each other.

### Limitation

- ❖ Qualitative data were collected through interviews, so the response to the practice may be inflated due to the social desirability bias of the respondents.
- ❖ This study may not show the temporal relationship between factors and dietary practice because it is a cross-sectional study.

## Data Availability

The dataset used and analyzed during the current study will be available from the corresponding author upon reasonable request.

## Abbreviations

ADA: American Diabetes Association
AOR: Adjusted Odd Ratio
CI: Confidence Interval
COR: Crude Odd Ratio
DM: Diabetes Mellitus
KM: Kilo Meter
SPSS: Statistical Package Of Social Science
WCUNEMMCSH: Wachemo University Nigist Eleni Mohamed Memorial Compressive Specialized Hospital
WHO: World Health Organization
